# Borderline personality disorder is associated with lower confidence in perception of emotional body movements

**DOI:** 10.3389/fpsyg.2014.01262

**Published:** 2014-11-04

**Authors:** Morten Kaletsch, Britta Krüger, Sebastian Pilgramm, Rudolf Stark, Stefanie Lis, Bernd Gallhofer, Karen Zentgraf, Jörn Munzert, Gebhard Sammer

**Affiliations:** ^1^Cognitive Neuroscience Group, Center for Psychiatry and Psychotherapy, Justus Liebig UniversityGiessen, Germany; ^2^Department of Psychology, Justus Liebig University GiessenGiessen, Germany; ^3^Institute for Sports Science, Justus Liebig University GiessenGiessen, Germany; ^4^Bender Institute of Neuroimaging, Justus Liebig UniversityGiessen, Giessen, Germany; ^5^Central Institute of Mental Health, Medical Faculty Mannheim/Heidelberg University, MannheimGermany; ^6^Institute for Sports Science, University of MunsterMünster, Germany

**Keywords:** borderline personality disorder, emotion perception, point–light displays, social cognition, embodiment, body movements, kinematics

## Abstract

Much recent research has shown that personality disorders are associated with an altered emotion perception. Whereas most of this research was conducted with stimuli such as faces, the present study examined possible differences in the perception of emotions expressed via body language and body movements. 30 patients with borderline personality disorder (BPD) and 30 non-patients observed video scenes of emotional human interactions conveyed by point–light displays, rated the depicted valence, and judged their confidence in this rating. Patients with BPD showed no altered emotion perception (i.e., no biased perception in either a negative or a positive direction). They did not perceive and evaluate depicted emotions as being more extreme than healthy controls. However, patients with BPD showed less confidence in their perception of depicted emotions, especially when these were difficult to identify. The findings extend insights on altered emotion perception in persons with BPD to include the field of body movements.

## INTRODUCTION

Borderline personality disorder (BPD) is a psychological condition characterized by interpersonal dysfunction and problems, unstable relationships, emotional dysregulation, suicidal behavior, impulsive aggressions such as verbal outbursts, and rapid switches between idealizing and devaluing relationships [American Psychiatric Association (APA), 2000; Lieb et al., 2004]. These dysfunctional thoughts and behaviors may bring patients into conflict with other people, may lead them to experience rejection and abandonment more often than non-patients (Stepp et al., 2009; Renneberg et al., 2011; Lis and Bohus, 2013).

One central feature of the interactional problems of BPD patients is their altered emotion perception. Despite a broad body of studies, findings on this topic are not always consistent. On the one hand, people with BPD are assumed to have negative evaluation styles and beliefs about themselves, others, and the world; and that they make negatively biased assessments, are overly sensitive, and pay more attention to negative stimuli. Especially when exposed to neutral or ambiguous stimuli, patients—compared to healthy controls (HCs)—evaluate others as being more negative, aggressive, or extreme [American Psychiatric Association (APA), 2000; Arntz and Veen, 2001; Sieswerda et al., 2005; Barnow et al., 2009; Domes et al., 2009; Baer et al., 2012; Daros et al., 2014]. For example, people with BPD have been shown to perceive neutral or ambiguous pictures or videos of faces or whole body displays in a negatively distorted way (Dyck et al., 2009; Baer et al., 2012), pay more attention to emotions such as anger but are equally sensitive when recognizing emotions (Domes et al., 2008; Robin et al., 2012; Hagenhoff et al., 2013).

On the other hand and in contrast to findings on deficient emotion recognition, the literature also reveals results where no differences occur and even evidence of better performance of BPD patients on emotion recognition processes (Lynch et al., 2006; Minzenberg et al., 2006; Domes et al., 2008; Merkl et al., 2010; Franzen et al., 2011; Unoka et al., 2011; Robin et al., 2012; Schilling et al., 2012; Schulze et al., 2013; Sieswerda et al., 2013).

In addition to research on emotion evaluation biases within this patient group, several studies have shown that people with BPD recognize emotions presented as pictures or videos of faces or body displays less accurately than controls, and that this accuracy decreases with increasing affect intensity. They recognize negative emotions, particularly anger, disgust, and sadness, less well than controls; show deficits in recognizing neutral facial expressions; and perceive and evaluate the personality traits of film characters as being more extreme (Levine et al., 1997; Veen and Arntz, 2000; Bland et al., 2004; Domes et al., 2009; Dyck et al., 2009; Merkl et al., 2010; Unoka et al., 2011; Arntz and ten Haaf, 2012; Robin et al., 2012; Daros et al., 2013).

In patients with BPD, clinical observations and practice let us presume a lack of confidence regarding recognizing emotion of other people. This often refers to emotions, which are expressed moderately without extreme intensity, facial expressions or gestures. Since patients often had been exposed to the experience of violence and strong negative emotions during their life (Zanarini, 2000; Lieb et al., 2004; Barnow et al., 2005) one may hypothesize that this way of expressing emotions is more familiar to patients than a quieter and more moderate way of expressing emotions. Literature on confidence in emotion perception among patients is not numerous. Schilling et al. (2012) showed that patients were significantly more confident in their decision while performing a theory of mind paradigm wherein they should identify the emotions of facial expressions. Also, patients were more confident compared to controls in a false-memory paradigm in which participants were asked to recognize details from scenes they had watched earlier (Schilling et al., 2013). Due to the small number of reports in this research area, we were interested in whether and to what extend patients would differ in their feelings of confidence during emotion perception.

It has to be noted that most research on emotion perception in patients with BPD has focused on emotions expressed via basic facial expression and prosody. Until now, few studies have used whole body displays or entire movie scenes or film clips showing one person or even two people in an interaction (Domes et al., 2009). However, in everyday interactions, people do not present static facial expressions but rather a complex set of expressions, gestures, and body language. When people perceive their interaction partners, they use multiple modalities to infer their emotional states. Thus, focusing on facial expressions probably neglects an important human system for expressing emotion: the human body and its movements. Human body movement can also convey emotions, and observers can infer the emotional state of an individual or of interacting persons solely from their movements, even when they are far away and their faces of are not clearly visible (de Gelder, 2006). Furthermore, body movements do not only provide information on a possible unspecific threat as facial expressions do. Body movements or postures of anger implicate a more specific physical threat, they bind attention and also they also give a direct cue regarding an adequate behavioral response (Johansson, 1973; Sevdalis and Keller, 2011; Atkinson et al., 2012; Ennis and Egges, 2012; Lorey et al., 2012; Barliya et al., 2013; Kret and de Gelder, 2013; Gelder and Hortensius, 2014). Moreover, social interaction and social context seems to facilitate the emotion recognition process, so that observing two people interacting rather than just one person alone also enhances the validation of perceived emotion and the perception of emotions and confidence in this perception. This emphasizes the relevance of social context for emotion perception (Clarke et al., 2005; Kret and de Gelder, 2010; Lorey et al., 2012).

To investigate the perception of emotional body movements while completely excluding facial information and other distracting variables, this study exploits the advantages of point–light displays (PLDs). These are recordings of the kinematics of a few dots placed on a model’s body. This technique has already been used to detect gender differences in the perception of emotional body language (Alaerts et al., 2011; Sokolov et al., 2011), or for the investigation of differences in emotion perception of various patients groups with somatic and psychological impairments (Pavlova, 2012). Henry et al. (2012), for example, found an impaired processing of socially relevant motions and emotions among patients with dementia. Nackaerts et al. (2012) showed a lower accuracy among persons with autism spectrum disorder whilst recognizing a person’s movements or emotional state and Kaletsch et al. (2014) discovered that patients with major depressive disorder rated emotional body movements more negatively and with higher confidence compared to HCs. Also for patients with schizophrenia deficits in emotion perception of biological and emotional body movements and facial expressions are discussed (Tomlinson et al., 2006; Spencer et al., 2013).

The advantage of using such highly simplified representations is that they provide only kinematic movement information. This ensures that the perception process is not influenced by confounding variables in the stimulus material such as attractiveness, sympathy, and other cultural aspects found in the complex and natural stimuli of faces or whole-body presentations (Hoffmann et al., 2010).

Against this background, we examined the possible differences between patients with BPD and HC. More precisely, we hypothesized that patients with BPD would (a) rate the emotional valence of the depicted interactions more negatively compared to HC, (b) differ in the experienced intensity of depicted emotions, especially when emotional scenes are negative, and (c) differ from controls in how confident they are about their ratings of depicted emotions.

## MATERIALS AND METHODS

### ETHICAL STATEMENT

The study was approved by the local ethics committee (local ethics commission, Department of Psychology and Sports Science, and local ethics committee of the Department of Medicine, Justus Liebig University Giessen). All participants gave their informed written consent to participate in the study in accordance with the Declaration of Helsinki.

### PARTICIPANTS

The total sample consisted of 60 adults: 30 patients receiving treatment at the Center of Psychiatry and Psychotherapy at the university hospital of the Justus Liebig University Giessen and 30 HC (see **Table 1** for descriptive statistics).

**Table 1:** Results of descriptive statistics and *t*-test for age, questionnaire results, rating of valence, intensity of rating, and confidence in rating by group.

	Healthy controls			Patients			
	*N*	*M*	SD	*N*	*M*	SD	*t*	*df*
Age	30	34.3	10.6	30	30.43	10.45	1.42	58
Beck Depression Inventory, BDI-II	30	4.27	3.05	30	30.93	12.22	-11.59***	58
Toronto Alexithymia Scale, TAS-26	30	2.12	0.39	29	3.23	0.59	-8.43***	57
Positive and Negative Affect schedule, PANAS	30	3.18	0.64	29	2.18	0.87	5.00***	57
Positive and Negative Affect schedule, PANAS	30	1.18	0.28	28	2.17	1.04	5.01***	56
Borderline Symptom list, BSL	–	–	–	23	0.06	0.06	–	–
Rating of valence	30	4.04	0.27	30	3.91	0.39	1.48	58
Intensity of rating	30	5.55	0.29	30	5.59	0.32	-0.50	58
Confidence in rating	30	9.29	0.98	30	8.52	0.95	3.03*	58

*p < 0.05, ***p < 0.001.

The 30 patients (27 female, mean age = 30.4 years, SD = 10.4, range = 18–68) were diagnosed with BPD according to DSM-IV criteria. Of these patients, 13 were taking antidepressants; 3 antipsychotics; 10 a combination of drugs (antidepressant and/or antipsychotic and/or sedative and/or mood stabilizer); and four patients were taking no medication at the time of the study.

Five patients met the criteria for another current mental disorder: major depression (*n* = 1), eating disorder (*n* = 3), posttraumatic stress disorder (*n*= 1). Eight met the criteria for more than one other mental disorder: major depression, posttraumatic stress disorder, and eating disorder (*n* = 1); major depression and posttraumatic stress disorder (*n* = 1); major depression and anxiety disorder (*n* = 1); major depression and attention deficit disorder with hyperactivity (*n* = 1); posttraumatic stress disorder and eating disorder (*n* = 3); and anxiety disorder and eating disorder (*n* = 1).

Diagnoses were conducted by experienced psychiatrists and clinical psychologists. Patients with present or previous neurological disease or trauma, alcohol or drug dependence, acute or chronic psychotic disorders, bipolar disorders, as well as other medical conditions that could influence cognitive functioning were not included in the study.

The 30 age-matched healthy adults (27 female, mean age = 34.3 years, SD = 10.6, range = 20–51) were recruited as a HC group. Controls were derived from a population of 96 healthy subjects, who were recruited as part of the project across different age groups and levels of education. Age difference between controls and patients was similar (**Table 1**). The same HC group was used for prior studies (Lorey et al., 2012; Kaletsch et al., 2014). The same exclusion criteria were applied as for patients. In addition, HC were excluded if they had any history of psychiatric or neurological disorders, any history or current use of psychoactive medication, or a score higher than 13 on the Beck Depression Inventory, BDI-II (Hautzinger et al., 2009). The following questionnaires in their German versions were administered to better describe our sample: Beck-Depression Inventory (BDI-II; Hautzinger et al., 2009), Toronto Alexithymia Scale (TAS-26; Kupfer et al., 2001), Positive and Negative Affect Schedule (PANAS; Watson et al., 1988), and only for patients with BPD the Borderline Symptom List (BSL; Bohus et al., 2001). For all questionnaires (except Borderline Symptom List) the scores differed statistically significant between groups (**Table 1**). Within each group, questionnaire results were not correlated to any of the dependent variables – and thus gave no further insight into how they are related or might have influenced the results.

### PRODUCING PLDs

The procedure for creating and validating stimuli was the same as that in Lorey et al. (2012; Kaletsch et al., 2014) where it is also described in more detail. Seven pairs of actors provided the movements for PLDs. Each pair was asked to perform an interaction portraying one of the following four emotions: anger, sadness, joy, and love. We pooled interactions with anger and sadness into the category “negative” and interactions with love and joy into the category “positive.” Prior to acting, we gave both actors a script instructing them to perform the same emotion together in order to produce a behavioral pattern that was as symmetrical as possible. We asked them to act out the emotion immediately, but gave them complete freedom to express the emotions in whatever way they liked—for example, by overt symbolic gestures. We produced at least four clips of each pair and each emotional scene. In addition, for each of the dyadic PLDs (scene with two actors: dyad), we created a monadic PLD version consisting of the dots of one of the two individuals alone (scene with one actor: monad). Apart from this, these monadic scenes still displayed the same emotion with the same movements. This resulted in a corpus of 96 recordings with eight recordings for each category (monad vs. dyad × positive vs. negative × 3 difficulty levels, see below).

**FIGURE 1:**
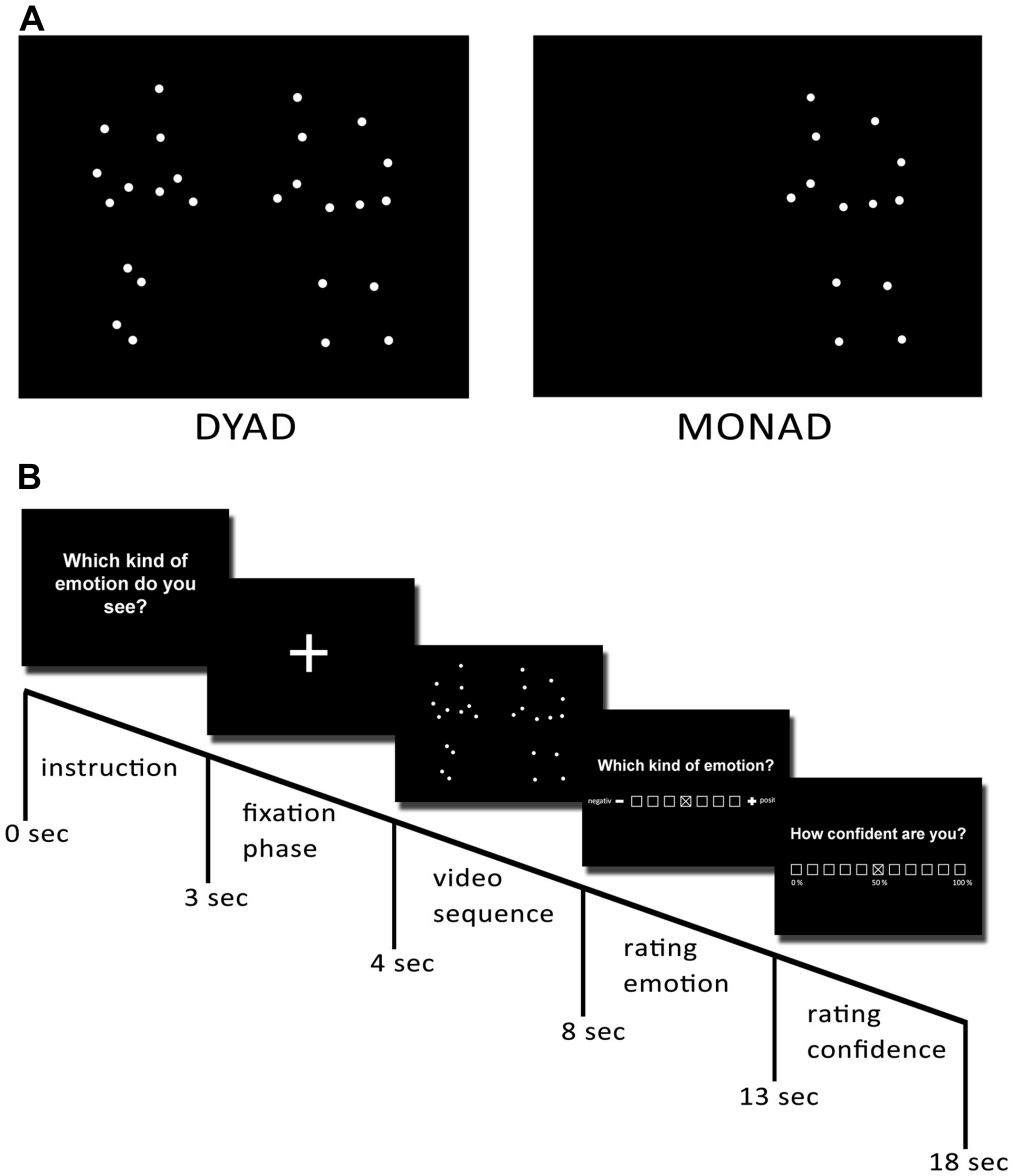
**Preparation of stimuli.** Point–light displays were created by attaching 13 reflective markers to an actor’s head, shoulders, elbows, wrists, hips, knees, and ankles. They were then tracked using a Vicon motion-capture system. **(A)** Examples of dyadic and monadic point–light displays. **(B)** Temporal structure of the paradigm. Reprinted from Kaletsch et al. (2014).

We attached 13 reflective markers to defined anatomical landmarks on the upper body (including the shoulders, the elbow joints, the wrists, and the forehead) and the lower body (including the hips, the knee joints, and the ankles) of each actor (**Figure 1**). Then we recorded all interactions with a 12-camera VICON MX system (Oxford Metrics, Oxford, England) operating at 100 Hz. After capturing, we postprocessed the data with Nexus 1.5.2 (Vicon Motion Systems, Oxford, England) in order to calculate 3-D coordinates of the markers. We created the video files in a two-step process using Matlab software (MathWorks, Natick, MA, USA). First, for each point in time, we plotted the 3-D coordinates of the 13 markers as white spheres on a black background. Then, we rendered the frames of the captured scenes as audio–video interleaved (avi) movie files at a frame rate of 25 Hz. For each scene, we created 4-s video files viewed from the front. In all presented PLDs, the dots appeared white against a black background at an approximate viewing distance of 50 cm (**Figure 1**).

### STIMULI: VALIDATION AND DETERMINATION OF DIFFICULTY OF EACH EMOTIONAL SCENE

Prior to the experiment, we determined an index of item difficulty for all recorded PLDs in order to separate the recordings into three classes (easy, medium, and difficult to recognize). We asked 30 participants who did not participate in the present study to evaluate the negativity or the positivity of the emotions displayed in the videos in a forced-choice paradigm. We created the three categories of item difficulty by calculating the percentage of people who agreed on the depicted emotion of the video scene. Thus, easy videos were defined by a consensus of 91–100%; medium videos, by a consensus of 71–90%; and difficult videos, by a consensus of 50–70%. For further description of the procedure see Lorey et al. (2012) and Kaletsch et al. (2014).

### PROCEDURE

Prior to the main experiment all participants completed questionnaires. Either before or after the actual experiment a control session was conducted, to ensure participants’ ability to recognize movements from PLDs. We presented participants with control stimuli depicting sports movements such as juggling and basketball, and asked them to give a brief definition of each movement as quickly as possible. One-half of the participants started with the experiment and the other half with the control session in order to control for sequence effects.

The experiment consisted of the aforementioned 96 video trials which were presented in a randomized order. Each single trial started with the instruction (3 s), followed by a gaze fixation phase (1 s), and the respective video sequence (4 s). After watching the video, participants were asked to assess the depicted emotional valence of the videos. Ratings were done using an on-screen 7-point scale ranging from 1 (*negative*) to 7 (*positive*) with 4 marking the neutral (neither positive nor negative emotion) center of the scale. The cursor was placed in the middle of the scale at the beginning of each rating. To control for a rating bias caused by the orientation of the scale, the scale was flipped for one-half of the participants. After each valence rating, participants were asked to report how confident they were about their rating on an 11-point on-screen scale ranging from 1 (0% *confidence*) to 11 (100% *confidence*).

### DATA ANALYSIS AND STATISTICS

For statistical data analysis all valence ratings were recorded. Scores of 1 to 3 always reflected a negative rating and scores of 5 to 7 a positive rating. For the statistical analysis, we calculated mean scores for each rating and each experimental condition. We calculated mean scores for the perceived valence by summing up all responses on the 7-point scales and dividing the sum by the number of videos displayed. We operationalized intensity of ratings as the extent to which participants gave ratings that were closer to the endpoints on the 7-point scale, under the assumption that intensity would be coded higher when the rating was closer to the ends of the scale. To create mean scores of intensity of ratings, we reversed all scores on the 7-point scale for ratings of perceived valence of negative videos (1 into 7, 2 into 6, etc.), to obtain scores comparable to those for positive videos. In other words, for both kinds of videos (negative and positive), higher scores indicated a higher intensity of ratings. We calculated mean scores on confidence by summing all responses from the 11-point scale and dividing the sum by the number of displayed videos.

To investigate differences between patients with BPD and HC in perceiving emotional valence, the perceived intensity of emotions, and confidence in emotion perception, repeated measurement ANOVAs were computed for perceived valence, intensity, and confidence. Main factors were the *depicted emotion* (positive vs. negative), the *social context* (monads vs. dyads), the *difficulty* of videos (easy, medium, difficult), and *group* as a categorical between-group factor. Means and standard deviations are displayed in **Table 1**, results of repeated measurement ANOVAs could be found in **Table 2**.

**Table 2:** Statistical data of depicted emotion × social context × difficulty repeated-measures ANOVA for rating of valence, intensity of rating, and confidence in rating.

	*df*	*F*	η^2^	*p*
**Rating of emotional valence**				
Group (between-group factor)	1, 58	2.19	0.04	0.14
Depicted emotion	1, 58	1792.99	0.96	0.000*
Depicted emotion × group	1, 58	0.25	0.00	0.61
Social context	1, 58	7.92	0.12	0.007*
Social context × group	1, 58	3.56	0.06	0.06
Difficulty	2, 116	10.09	0.15	0.000*
Difficulty × group	2, 116	0.19	0.00	0.82
Depicted emotion × social Context	1, 58	514.41	0.89	0.000*
Depicted emotion × social context × group	1, 58	1.478	0.03	0.23
Depicted emotion × difficulty	2, 116	258.92	0.82	0.000*
Depicted emotion × difficulty × group	2, 116	0.02	0.00	0.98
Social context × difficulty	2, 116	17.34	0.23	0.000*
Social context × difficulty × group	2, 116	0.32	0.00	0.73
Depicted emotion × social context × difficulty	2, 116	15.36	0.21	0.000*
Depicted emotion × social Context × difficulty × group	2, 116	0.43	01	0.65
**Intensity of ratings**				
Group (between-group factor)	1, 58	0.25	0.00	0.62
Depicted emotion	1, 58	0.31	0.01	0.58
Depicted emotion × group	1, 58	2.19	0.04	0.14
Social context	1, 58	514.41	0.89	0.000*
Social context × group	1, 58	1.48	0.03	0.23
Difficulty	2, 116	258.92	0.82	0.000*
Difficulty × group	2, 116	0.02	0.00	0.98
Depicted emotion × social context	1, 58	7.92	0.12	0.01*
Depicted emotion × social context × group	1, 58	3.56	0.06	0.06
Depicted emotion × difficulty	2, 116	10.09	0.15	0.000*
Depicted emotion × difficulty × group	2, 116	0.19	0.00	0.82
Social context × difficulty	2, 116	15.36	0.21	0.000*
Social context × difficulty × group	2, 116	0.43	0.01	0.65
Depicted emotion × social context × difficulty	2, 116	17.34	0.23	0.000*
Depicted emotion × social Context × difficulty × group	2, 116	0.32	0.00	0.72
**Confidence in rating**				
Group (between-group factor)	1, 58	9.20	0.14	0.000*
Depicted emotion	1, 58	7.67	12	0.01*
Depicted emotion × group	1, 58	0.21	0.00	0.65
Social context	1, 58	147.82	0.72	0.000*
Social context × group	1, 58	0.86	0.02	0.36
Difficulty	2, 116	112.79	0.66	0.000*
Difficulty × group	2, 116	3.04	0.05	0.05
Depicted emotion × social context	1, 58	8.32	0.13	0.01*
Depicted emotion × social context × group	1, 58	3.37	0.06	0.07
Depicted emotion × difficulty	2, 116	3.49	0.06	0.03*
Depicted emotion × difficulty × group	2, 116	0.33	0.01	0.72
Social context × difficulty	2, 116	12.20	0.17	0.000*
Social context × difficulty × group	2, 116	0.65	0.01	0.52
Depicted emotion × social context × difficulty	2, 116	17.69	0.23	0.000*
Depicted emotion × social Context*difficulty*group	2, 116	1.75	0.03	0.18

Analysis of variance; *p< 0.05.

All statistics were calculated using SPSS software (Versions 19 and 20). An alpha level of 0.05 was used for all statistical tests. Since the approach in this work is innovatory in the field of emotion perception in patients with BPD, we will also report results with a significance level of 0.05–0.10. Thus, we ensure to give a more informative overview what might be important for future research.

## RESULTS

### CONTROL DATA

#### Control session: biological motion recognition test

Participants were able to identify each of the actions reliably and far above chance level. On average, 93.07% (range: 67–100%) of identifications were correct.

#### Influence of group on rated valence (negative or positive)

There was no significant effect of *group* (BPD vs. HC) on the rating of perceived emotional valence, *F*(1,58) = 2.19, *p* = 0.144. Therefore, patients with BPD did not differ from HC in rating valence.

The two-way interaction between *social context* and *group* which was on the border of significance indicated that patients with BPD rated monadic scenes, but not dyadic scenes, more negatively than HC, *F*(1,58) = 3.56, *p* = 0.064. All results of the repeated measures ANOVAs are reported in **Table 2**. None of the two-, three-, or four-way interactions (*depicted emotion, social context, difficulty*) with *group* became statistically significant.

### INFLUENCE OF GROUP ON INTENSITY OF RATINGS

Regarding the intensity of participants’ ratings on depicted emotion, ANOVAs revealed no significant main effect of *group*, *F*(1,58) = 0.25, *p* = 0.616. Patients with BPD did not differ from HC in intensity of ratings.

We found a tendency in the non-significant three-way interaction between *social context, depicted emotion*, and *group* indicating that patients with BPD rated monadic scenes, but not dyadic scenes as being more intense than HC did, but only if the depicted emotion was negative, *F*(1,58) = 3.56, *p* = 0.064. The four-way interaction (*depicted emotion, social context, difficulty*) with *group* was not significant.

### INFLUENCE OF GROUP ON CONFIDENCE IN RATINGS

**FIGURE 2:**
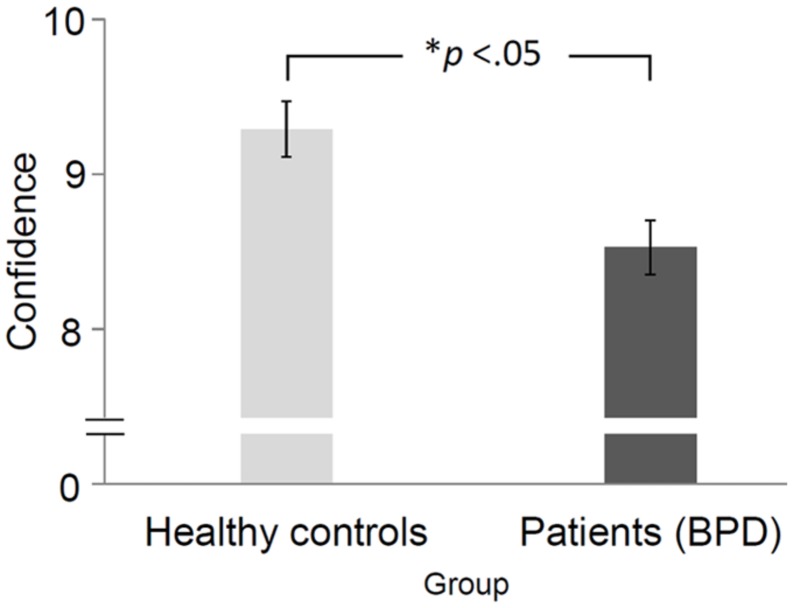
**Average confidence ratings and standard deviations of healthy controls (HCs) and patients with BPD.** Error bars represent standard error of mean. *The difference was significant at the 0.05 level.

There was a significant main effect of group membership on confidence in the rating of perceived emotional valence *F*(1,58) = 9.20, *p* = 0.004. Patients with BPD rated depicted emotions less confidently than HC (**Figure 2**).

Additionally, we found a tendency in the non-significant two-way interaction between *difficulty* and *group,* indicating that patients with BPD became less confident about their ratings as the depicted emotion became more difficult to recognize, *F*(2,116) = 3.04, *p* = 0.052. None of the three- or four-way interactions (*depicted emotion, social context, difficulty*) with *group* were significant.

## DISCUSSION

The present study was conducted to investigate differences in the perception of emotional body movements in patients with BPD. Body language is a reliable source of information for assessing emotional states. It can convey emotions and may trigger more complex behavioral responses within the observer than facial expressions might do (de Gelder, 2006). Thereby it plays a significant role in the context of social interacting and functioning which are domains in which many psychiatric patients encounter difficulties. However, until now, no study using PLDs has examined the relationship between BPD and the perception of emotions presented solely through body movements without additional facial expressions.

This study provided one major finding: In comparison to HC, patients with BPD felt less confident when evaluating the depicted emotional scenes. Although not statistically significant, however, due to the exploratory nature of this work it is still noteworthy that patients tended to become less confident the more difficult the videos became to evaluate. Also not statistically significant but still noteworthy was that there might be the effects that firstly, patients with BPD rated monadic but not dyadic scenes more negatively; and secondly, patients rated monadic scenes as being more intense. These indications could be investigated in a subsequent study. To sum up, the present work provides evidence that patients with BPD differ in their feelings of confidence during perception of and response to emotional body movements when they are presented within a PLD paradigm. Less confidence in emotion perception may lead patients to refrain from approaching social situations or interactions or to behave less confidently within such situations. This behavior, in turn, could have adverse effects on such or future interactions.

When examining the first hypothesis that patients with BPD would show a general bias when rating the emotional valence of the depicted interactions, we found no differences between groups. Thus, patients rated the emotions shown just as well as HC. This was also true in regard to the factors difficulty, social context and depicted emotion, which not lead to any significant group differences or interaction effects. Thus, and in contrast to findings that reflect a biased or distorted emotion perception, our findings are similar to those that patients did not show general deficits in emotion recognition or a perception bias, especially toward negative emotions (Minzenberg et al., 2006; Domes et al., 2008; Unoka et al., 2011; Robin et al., 2012; Schilling et al., 2012; Sieswerda et al., 2013). Contrary to previous findings of a negatively biased perception of emotions in patients with BPD (Arntz and Veen, 2001; Barnow et al., 2009; Dyck et al., 2009; Baer et al., 2012) we were unable to detect such a clear effect in this study as that found when applying the same paradigm to a group of patients with major depressive disorder (Kaletsch et al., 2014). Whereas patients with major depressive disorder may tend to perceive emotional interactions with a negative bias on a more general level in line with a negative view of the world, people with BPD might show differential effects not when observing but only when directly involved in an interaction and therefore making the interaction more personally relevant.

Even though it failed to attain significance, the two-way interaction between social context and group indicated that patients with BPD rated monadic scenes but not dyadic scenes more negatively than HC. This effect, however, only indicated a trend and future research could investigate the relationship between aversive feelings when being lonely, empathy, and the number of persons observed.

We also could not confirm our second hypothesis that patients with BPD would perceive emotional interactions as being more intense than HC would. This is in line with research reporting an inability to detect a dichotomous or extremely intense evaluation style in BPD patients (Veen and Arntz, 2000; Sieswerda et al., 2013). Contrary to previous findings of a more extreme evaluation of personality traits we did not detect such an effect in this study as that found when applying the same paradigm to a group of patients with major depressive disorder (Kaletsch et al., 2014). Whereas patients with major depressive disorder may tend to perceive negative emotional interactions more intense, people with BPD might show differential effects not when observing but only when directly involved in an interaction and therefore making the interaction more personally relevant. The non-significant interaction for the intensity of ratings indicated that patients with BPD rated monadic scenes as being more intense, but only when the depicted emotional scene was negative.

The most important finding in the present study is that patients with BPD were significantly less confident when perceiving and evaluating the depicted emotional scenes. This lack of confidence in perceiving emotional body movements may be based on a history of invalidating, instable, and confusing emotional experiences gained with, for example, attachment figures who provided unreliable emotional responses. This insecurity, in turn, could lead to less confidence in evaluating personal interactions (Fonagy, 2000). Such a pattern of emotion perception combined with a high need for self-protection could lead to insecure or less competent behavior within social interactions, to withdrawal, or to avoidance behavior (Newman et al., 2007). In the long term, feelings of insecurity may lead to lower self-confidence and self-efficacy, negative and instable affect, or states of emotional dysregulation (Johnson et al., 2002; Fruzzetti et al., 2005).

Interestingly, even though it failed to attain significance the two-way interaction between difficulty and group indicated that patients with PBD became less confident as the depicted emotion became more difficult to recognize. This could imply that patients are more confident when depicted emotions are strong and expressive, as they often experience them to be for themselves, or people in interactions with patients often might have expressed them, but become less confident, the more subtle and sensitive the expressed emotions are. This might be understood against the background that patients often have a history of violence, abuse and extreme emotions in the past, so that they might be more familiar with this kind of emotion information and therefore might have difficulties in assessing ambiguous, difficult or rather less extreme emotions (Zanarini, 2000; Lieb et al., 2004; Barnow et al., 2005).

Contrary to our results Schilling et al. (2012, 2013) found that patients with BPD showed more confidence than controls when completing the Reading the Mind in the Eyes Test (Baron-Cohen et al., 2001) and during a false memory paradigm whose stimuli differed greatly from those used in the present experiment. However, they assessed confidence in a similar way to our paradigm only in the Reading the Mind in the Eyes Test, and compared their answers only within the highest confidence category between groups, whereas, in our analysis, we compared mean confidence values. Perhaps patients are much less confident when observing a social and emotional interaction compared to a static image. A comparison study could shed light on this issue. Schilling et al. (2012) discussed a possible overconfidence bias or a possible tendency to respond in extreme ways. We could not find evidence for either an overconfidence bias or en extreme response or evaluation style while rating emotional valence or in regard to intensity of ratings.

### LIMITATIONS AND FURTHER DIRECTIONS

A limitation of this study is that our findings are not undoubtedly specific for emotion recognition tasks but are linked to overall reduced feelings of confidence in patients with BPD while performing the task. In subsequent studies this effect should be investigated by including a non-emotional recognition task as control condition. If the effect turns out to be specific, it would be necessary to examine why and under what conditions patients show this lack of confidence and how it is acquired. Further research should investigate the possible consequences of an uncertain perception of emotional body movements in personal interactions and how this may contribute to the perpetuation of BPD. How does lower confidence influence social interactions? How does it influence a patient’s self-concept and how, if these effects are harmful, can it be addressed in a therapeutic context? Another interesting approach is to adapt the paradigm so that the proband gets addressed by a counterpart and thereby would be directly involved into the interaction.

Due to the novelty of this approach to asses emotion perception processes within clinical samples using PLDs, we also have reported and mentioned non-significant results on the border of significance, which may provide useful information and may be further investigated in subsequent studies. It could be a lack of power to be responsible for the missing statistical significance, which could be adjusted in subsequent studies by enlarging the relevant samples.

## CONCLUSION

This is the first study to investigate differences between patients with BPD and HCs when perceiving emotions expressed solely via body movements conveyed by PLDs. First and foremost, we have demonstrated that patients with BPD are less confident when perceiving and evaluating emotional scenes, and that their confidence declines the more difficult the emotional scenes become. Additionally, unlike patients with major depressive disorder (Kaletsch et al., 2014), BDP patients do not reveal an altered emotion perception; that is, any biased perception in either a negative or a positive direction. However, they do show a tendency to evaluate monadic stimuli more negatively. Further, in general, BDP patients do not perceive and evaluate depicted emotions more extremely in the sense of a dichotomous thinking but tend to evaluate monadic scenes as being more intense when the depicted emotion is negative.

## Conflict of Interest Statement

The authors declare that the research was conducted in the absence of any commercial or financial relationships that could be construed as a potential conflict of interest.
